# Impact of Remineralization Profile Shape on the Air‐Sea Carbon Balance

**DOI:** 10.1029/2020GL091746

**Published:** 2021-04-09

**Authors:** Jonathan Maitland Lauderdale, B. B. Cael

**Affiliations:** ^1^ Department of Earth, Atmospheric and Planetary Sciences Massachusetts Institute of Technology Cambridge MA USA; ^2^ Ocean Biogeosciences National Oceanography Centre Southampton UK

**Keywords:** atmospheric CO_2_, biological pump, carbon cycle, export fluxes, Martin Curve, structural uncertainty

## Abstract

The ocean's “biological pump” significantly modulates atmospheric carbon dioxide levels. However, the complexity and variability of processes involved introduces uncertainty in interpretation of transient observations and future climate projections. Much research has focused on “parametric uncertainty,” particularly determining the exponent(s) of a power‐law relationship of sinking particle flux with depth. Varying this relationship's functional form introduces additional “structural uncertainty.” We use an ocean biogeochemistry model substituting six alternative remineralization profiles fit to a reference power‐law curve, to systematically characterize structural uncertainty, which, in atmospheric pCO_2_ terms, is roughly 50% of parametric uncertainty associated with varying the power‐law exponent within its plausible global range, and similar to uncertainty associated with regional variation in power‐law exponents. The substantial contribution of structural uncertainty to total uncertainty highlights the need to improve characterization of biological pump processes, and compare the performance of different profiles within Earth System Models to obtain better constrained climate projections.

## Key Points

- Six alternative flux profiles fit to a Martin Curve yield large differences in atmospheric carbon
- Structural uncertainty comprises one‐third of total uncertainty in the ocean's biological pump
- Varying particle attenuation with depth may account for half of the biological pump's overall carbon drawdown

## 1 Introduction

Carbon and nutrients are consumed by phytoplankton in the surface ocean during primary production, leading to a downward flux of organic matter. This “marine snow” is transformed, respired, and degraded by heterotrophic organisms in deeper waters, ultimately releasing those constituents back into dissolved inorganic form. Oceanic overturning and turbulent mixing return resource‐rich deep waters back to the sunlit surface layer, sustaining global ocean productivity. The “biological pump” maintains this vertical gradient in nutrients through uptake, vertical transport, and remineralization of organic matter, storing carbon in the deep ocean that is isolated from the atmosphere on centennial and millennial timescales, lowering atmospheric CO_2_ levels by hundreds of micro‐atmospheres (Ito & Follows, 2005; Volk & Hoffert, 1985). The biological pump resists simple mechanistic characterization due to the complex suite of biological, chemical, and physical processes involved (Boyd et al., 2019), so the fate of exported organic carbon is typically described using a depth‐dependent profile to evaluate the degradation of sinking particulate matter.

Various remineralization profiles can be derived from assumptions about particle degradability and sinking speed(s) (Armstrong et al., 2001; Banse, 1990; Cael & Bisson, 2018; Kriest & Oschlies, 2008; Lutz et al., 2002; Martin et al., 1987; Middelburg, 1989; Rothman & Forney, 2007; Suess, 1980). The ubiquitous “Martin Curve” (Martin et al., 1987) is a power‐law profile (Equation 1) that assumes slower‐sinking and/or labile organic matter is preferentially depleted near the surface causing increasing sinking speed and/or remineralization timescale with depth (e.g., Kriest & Oschlies, 2011).

(1) $$f_{p}\left(z\right)=C_{p}z^{-b},$$

where *f*_*p*_(*z*) is the fraction of the flux of particulate organic matter from a productive layer near the surface (Buesseler et al., 2020) sinking through the depth horizon *z* [m], *C*_*p*_ [m^*b*^] is a scaling coefficient, and *b* is a nondimensional exponent controlling how *f*_*p*_ decreases with depth. Equation 1 is often normalized to a reference depth *z*_*o*_ but this parameter is readily absorbed into *C*_*p*_.

Considerable effort has been dedicated to determining value(s) for the exponent, *b* (e.g., Berelson, 2001; Gloege et al., 2017; Henson et al., 2012; Honjo et al., 2008; Kriest et al., 2012; Kwon & Primeau, 2006; Martin et al., 1987, 1993; Primeau, 2006; Wilson et al., 2019). Open ocean particulate flux observations from the North Pacific (Martin et al., 1987) indicate a *b* value of 0.858. Further analyses of expanded sediment trap datasets suggest a possible range of approximately 0.84 ± 0.14 for the global *b* value (Berelson, 2001; Gloege et al., 2017; Honjo et al., 2008; Martin et al., 1993; Primeau, 2006), though a much wider range has been observed when including regional variability in *b* and optically and geochemically derived flux estimates (Guidi et al., 2015; Henson et al., 2012; Pavia et al., 2019). This may result from differences in temperature (Matsumoto, 2007), microbial community composition (Boyd & Newton, 1999), particle composition (Armstrong et al., 2001), oxygen concentration (Devol & Hartnett, 2001), particle aggregation (Gehlen et al., 2006; Niemeyer et al., 2019; Schwinger et al., 2016), or mineral ballasting (Gehlen et al., 2006; Pabortsava et al., 2017).

Uncertainty in the value of *b* translates to uncertainty in the biological pump's impact on the ocean carbon sink, atmosphere‐ocean carbon partitioning, and climate model projections. Thus, constraining *b* for the modern ocean and how it may differ in the past, or the future, is of much interest from a climate perspective. Varying a global value of *b* between 0.50 and 1.4 altered atmospheric pCO_2_ by 86–185 μatm after several thousand years of equilibration, in an influential modeling study (Kwon et al., 2009): Higher values of *b* result in enhanced particle remineralization at shallower depths. Shallow water masses are more frequently ventilated, allowing remineralized CO_2_ to be released back into the atmosphere on shorter timescales. Due to this depth‐dependence, a small change of degradation depth can appreciably change atmospheric pCO_2_ (Kwon et al., 2009; Yamanaka & Tajika, 1996). Varying *b* over the plausible range in global values between 0.70 and 0.98 produces a more modest change in atmospheric pCO_2_, over the range of (−16, +12)μatm (Gloege et al., 2017), while the modeled uncertainty in atmospheric pCO_2_ associated with regional variation in *b* is estimated between 5 and 15 μatm (Wilson et al., 2019).

Biogeochemical models are subject not only to parametric uncertainty (which value for *b* and how *b* varies in space and time), but also structural uncertainty, that is, which equation(s) to choose for the vertical flux of organic matter. The Martin Curve power‐law is an empirical fit to sediment trap data, but several other functional forms have also been put forward (Armstrong et al., 2001; Banse, 1990; Dutkiewicz et al., 2005; Lutz et al., 2002; Marsay et al., 2015; Middelburg, 1989; Rothman & Forney, 2007; Suess, 1980) that fit sediment trap fluxes equivalently well and have equal if not better mechanistic justification (Cael & Bisson, 2018). Atmospheric pCO_2_ and many other global biogeochemical properties (Aumont et al., 2017; Kriest et al., 2012; Kwon & Primeau, 2006) will be affected by this structural uncertainty, so it is critical to evaluate the impact of choosing one remineralization profile “shape” over another.

We assess the effect of remineralization profile shape on biological pump strength and evaluate a comprehensive estimate of structural uncertainty in terms of atmosphere‐ocean carbon partitioning in a global ocean biogeochemistry model. We substitute the reference power‐law curve for six plausible alternative remineralization profiles: exponential (Banse, 1990; Dutkiewicz et al., 2005; Gloege et al., 2017; Marsay et al., 2015), ballast (Armstrong et al., 2001; Gloege et al., 2017), double exponential (Lutz et al., 2002), stretched exponential (Cael & Bisson, 2018; Middelburg, 1989), rational (Suess, 1980), and upper incomplete gamma function of order zero (Rothman & Forney, 2007, we use the shorthand “gamma function” for “upper incomplete gamma function of order zero,” although different orders are possible). Each form corresponds to a basic mechanistic description of particle fluxes (Cael & Bisson, 2018), that we tightly constrained to the reference profile by statistically minimizing export fraction misfits or by matching degradation depth scales (Kwon et al., 2009). See Supporting Information for derivations of these profiles.

These simulations indicate that structural uncertainty is an appreciable component, around one‐third, of total uncertainty for understanding the biological pump (with the remaining two‐thirds attributed to parametric uncertainty in *b*). Changing remineralization functional form alters atmospheric pCO_2_ by ∼10–15 μatm depending on how structural uncertainty is quantified, equivalent to ∼0.08 uncertainty in a global value of the power‐law exponent, *b*, and similar to the uncertainty resulting from regional variation of *b*.

Our results underscore the importance of characterizing basic mechanisms governing the biological pump. Furthermore, our results corroborate that depth‐dependence of these mechanisms is particularly important (Gehlen et al., 2006; Kriest & Oschlies, 2008): not only is biological pump‐driven carbon export and storage an important control on atmospheric pCO_2_, we find that rapidly decreasing particle degradation in the upper ocean is equally important for a sufficient quantity of carbon to become isolated in the deep ocean. While a given flux curve may be chosen for historical reasons or mathematical convenience, its skill should be compared to those of other idealized flux profile parameterizations in Earth System Models used for projections of future climate.

## 2 Materials and Methods

### 2.1 Fitting the Alternative Remineralization Curves

**Figure 1:**
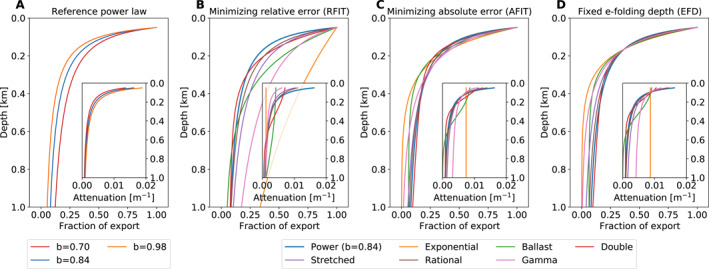
Fraction of sinking particulate organic matter exported from the 50 m surface layer remaining at each depth for (a) the reference power‐law (Equation 1) with exponents 0.84 ± 0.14, and six alternative functions (Equations S1–S6) fit to the reference power‐law curve (*b* = 0.84) by (b) statistically minimizing the relative error (“RFIT”), or (c) the absolute error (“AFIT”), and (d) matching the e‐folding depth scale of 164 m (“EFD”). See Section 2, Table S1 for fitting details, coefficients, and fit statistics. Inset plots show the attenuation rate of the export flux with depth $\left[\frac{1}{f}\frac{\partial f}{\partial z},m^{-1}\right]$.

We fit the alternative functions for export fluxes and remineralization (Figure 1, Equations S1–S6, see Supporting Information) to the reference power‐law curve (Equation 1) with the exponent *b* = 0.84 using nonlinear regression on the model vertical grid to minimize the absolute curve mismatch (“ABS” simulations). Subsurface points were weighted equally (1.0), except for a heavily weighted top‐level (valued 1,000, but the overall fit was largely insensitive to the choice of this value) to ensure all the profiles pass through the same value as the control profile, that is, fraction of export from the productive surface layer is unity. We further matched the e‐folding depth of remineralization to the reference (“EFD” simulations) by adding a second heavily weighted point to the reference power‐law at 164‐m depth (*z*_0_ *e* ^(1/*b*)^), with an export fraction of *e* ^−1^. In a third set (“RFIT” simulations), the nonlinear regression is performed on the natural logarithm of the remineralization fraction to minimize the relative error of the reference profile match. Goodness of fit is evaluated by the Standard Error of Regression, S, which is the sum of squared residuals, divided by statistical degrees of freedom (number of points minus number of parameters). Coefficients and S values for the 18 curves are given in Table S1.

### 2.2 Numerical Ocean Biogeochemistry Model

Alternative remineralization profiles are substituted into global ocean simulations of a coarse resolution (2.8°, 15 vertical levels) configuration of the Massachusetts Institute of Technology general circulation model, MITgcm (Marshall et al., 1997), coupled to an idealized marine biogeochemistry model that considers the coupled cycles of dissolved inorganic carbon, alkalinity, phosphate, dissolved organic phosphorus, oxygen, and dissolved iron (Dutkiewicz et al., 2006; Parekh et al., 2005, 2006).

Two‐thirds of surface net community production (that depends on light, phosphate, and iron using Michaelis‐Menten kinetics) is channeled into dissolved organic matter that is largely remineralized in the surface ocean with a timescale of 6 months (Yamanaka & Tajika, 1997), while one‐third is exported to the ocean interior via sinking particulate organic matter subject to depth‐dependent remineralization rates. Elemental biological transformations are related using fixed stoichiometric ratios $R_{C:N:P:Fe:O_{2}}=117:16:1:4.68\times 10^{-4}:-170$ (Anderson & Sarmiento, 1994) with a prescribed inorganic to organic rain ratio of 7% (Yamanaka & Tajika, 1996). The total atmosphere‐ocean carbon inventory is conserved as there is no riverine carbon input or sediment carbon burial, which may impact the model's transient behavior and steady state (Roth et al., 2014). Atmosphere‐ocean exchange of CO_2_ captures the magnitude and variation of observed air‐sea fluxes (Lauderdale et al., 2016).

Our model includes tracers to separate the in situ concentrations of carbon into (i) a component subducted from the surface layer and transported conservatively by ocean circulation (the “preformed” carbon concentration, *C*_*pre*_), and (ii) a component that integrates export and remineralization of sinking particles as a water mass transits the ocean interior (the “biological” carbon concentration, *C*_*bio*_), which encompasses both soft tissue regeneration and carbonate dissolution, and connects more directly to the biological pump (Ito & Follows, 2005; Volk & Hoffert, 1985). We integrate simulations for 10,000 years toward steady state in atmosphere‐ocean carbon partitioning.

## 3 Results

### 3.1 Varying the Exponent of the Reference Power‐Law Curve

Global power‐law exponent, *b*, estimates range from 0.70 (Primeau, 2006) based on sediment traps to ∼1.00 based on inverse models fit to tracer distributions (Kwon & Primeau, 2006, 2008; Kwon et al., 2009; Kriest et al., 2012). These values match the global *b* interquartile range of 0.70–0.98 in Gloege et al. (2017). We integrate three simulations with *b* = 0.84 ± 0.14 (Figure 1a) using the standard power‐law parameterization (Equation 1) to produce a baseline estimate of biological pump parametric uncertainty. The reference simulation has the exponent *b* = 0.84.

**Figure 2:**
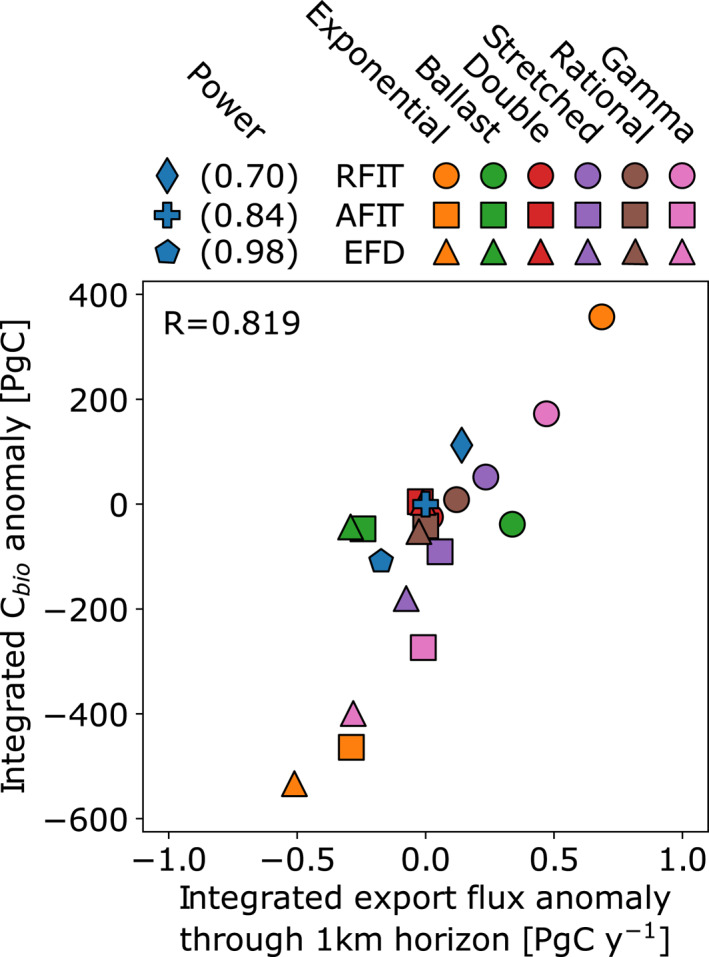
Change in the integrated export flux rate [PgC y^−1^] passing through the 1‐km depth level against integrated biological carbon reservoir anomaly [PgC], both with respect to the power‐law curve where *b* = 0.84 (Martin et al., 1987). Three power‐law simulations (*b* = 0.84 ± 0.14) are indicated by the blue symbols (diamond, cross, and pentagon), circle, square, and triangle symbols indicate that profile coefficients (Equations S1–S6) were derived by minimizing the relative fit error (“RFIT”), minimizing the absolute fit error (“AFIT”), and fixing the e‐folding depth of remineralization (“EFD”), respectively, to the reference power‐law curve. Values are given in Tables S2 and S3.

Higher *b* values cause the fraction of sinking particulate matter to decrease faster with depth, that is, attenuation $\left(1/f_{p}\cdot \partial f_{p}/\partial z\right)$ is higher in the upper ocean, whereas lower exponents have less attenuation and a larger proportion of export reaching the deep ocean (Figures 1a and S2a–S2f). A negative feedback occurs near the surface in our simulations. For example, when *b* is increased, higher rates of upper ocean attenuation cause an increase in surface nutrient availability, and therefore more overall biological production (see Δ*B*_*C*_, Table S2). Local biological activity enhancement increases local rates of particle export, evaluated by integrated fluxes through the deepest mixed layer depth (Δ*E*_*mld*_, Table S2). However, higher shallow export is compensated by greater upper ocean remineralization, due to larger exponent value, resulting instead in reduced export flux anomalies through 1‐km depth (Δ*E*_1*km*_, Table S2), and vice versa when *b* is decreased (e.g., global experiments in Kwon et al. [2009] and Kriest and Oschlies [2011]). The global ocean reservoir of biological carbon changes proportionally with Δ*E*_1*km*_ (Figure 2, blue symbols, S2g–S2l, and Δ*C*_*bio*_, Table S2) and inversely proportional to Δ*E*_*mld*_ (Figure S3a).

### 3.2 Impact of Alternative Remineralization Curve Shape

Generally speaking, the six alternative remineralization profiles (Equations S1–S6) objectively characterized by statistically fitting parameters to match the reference power‐law curve (*b* = 0.84) do reproduce similar sinking particle remineralization rates (Figures 1b–1d). This is perhaps not a surprise, since we would not consider these functions to be plausible alternatives to the Martin Curve if they could not describe export fluxes at least as well as a power‐law.

Nevertheless, the simple exponential and gamma function curves do not fit the reference power‐law profile as well as the other functions (Figures 1b–1d) because these profiles cannot capture a strong depth‐change in remineralization. The ballast profile has a more complex distribution of biological carbon anomalies in surface, intermediate, and deep waters such that the relationship between export flux and Δ*C*_*bio*_ is better captured by considering deeper horizons (e.g., green symbols in Figure 2 at the 1‐km horizon vs. 2 km in Figure S3b).

Simulations with lower‐attenuation profiles result in increased export fluxes (Figure S4), and vice versa, as with the simulations varying *b* (Figure 2). These particulate flux anomalies translate into changes in the distribution of biological carbon, with positive export flux anomalies through the 1‐km depth horizon (Δ*E*_1*km*_) corresponding to an increase in the biological carbon pool (*C*_*bio*_, Figure 2), while negative export flux anomalies result in lower biological carbon concentrations. For instance, in RFIT simulations, the exponential and gamma function profiles show an increase in 1 km export fluxes and biological carbon storage, while the reverse occurs for exponential and gamma profiles in AFIT and EFD simulations.

Geographically, stronger ocean interior sinking fluxes tend to redistribute biological carbon into the Southern Ocean and deep North Pacific at the expense of the North Atlantic (Figures S5–S7), while shallower remineralization tends to increase North Atlantic biological carbon concentrations whilst decreasing concentrations in the Southern Ocean and deep North Pacific. This is a reflection of the accumulation of *C*_*bio*_ as a water mass transits the global meridional overturning circulation with the oldest waters upwelling in the Southern Ocean and North Pacific (Kriest et al., 2012; Kriest & Oschlies, 2011; Kwon et al., 2009; Kwon & Primeau, 2006; Romanou et al., 2014). These anomalies of *C*_*bio*_ (Figures S5–S7) account for the direct effects of organic and inorganic particle fluxes. At the same time, changes in biological activity affect surface alkalinity both through carbonate export and surface charged nutrient abundance, which reinforces ocean carbon uptake or outgassing due to the inverse relationships relating carbon and alkalinity to CO_2_ solubility (Kwon et al., 2009). However, atmospheric CO_2_ anomalies driven by different remineralization profiles integrate several compensating processes. Indirect carbon changes, including the effect of alkalinity on ocean carbon saturation, regenerated carbon upwelling, as well as unrealized air‐sea exchange due to the finite timescale of atmosphere‐ocean CO_2_ fluxes (Ito & Follows, 2005; Lauderdale et al., 2013, 2017), that are captured by preformed carbon anomalies actually counteract approximately two‐thirds of the direct biological ocean carbon storage.

### 3.3 Evaluating Structural Uncertainty of the Biological Pump

**Figure 3:**
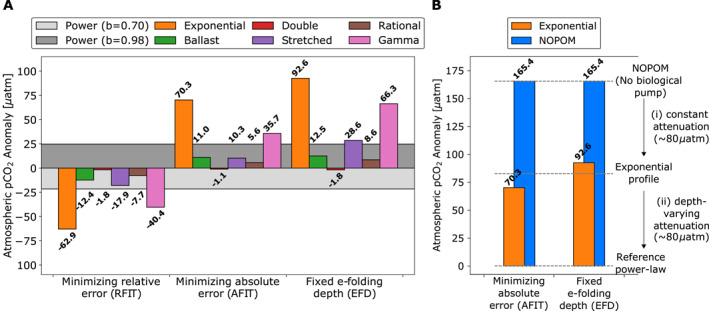
Impact of alternative remineralization curve shape on the air‐sea carbon balance (a) atmospheric pCO_2_ anomalies (μatm) for remineralization profiles with respect to the reference power‐law (*b* = 0.84) for power‐law exponent values *b* = 0.70 and 0.98, and statistical fits of alternative profiles “RFIT” (left), “AFIT” (middle), and “EFD” (right). Values are given in Tables S2 and S3; (b) comparison of a simulation with no particulate organic matter production (“NOPOM”), that is., no biological pump, to the simple exponential profile, and reference power‐law profile for “AFIT” (left), and “EFD” (right) fits. From a “NOPOM” ocean without sinking particle export, establishing (i) a biological pump with an exponential remineralization curve and constant attenuation of sinking particles with depth only draws down roughly 80 μatm atmospheric CO_2_, while a further 80 μatm drawdown can be achieved by establishing (ii) a biological pump with a power‐law remineralization profile that has decreasing particle attenuation, or increasing remineralization length scale, with depth. Thus, biological pump non‐linearity appears to be equally important for air‐sea carbon partitioning as export and storage of biological carbon.

Altering the strength of the biological pump leads to changes in air‐sea carbon balance. The reference simulation has a steady‐state atmospheric pCO_2_ of 269.3 μatm. Increasing *b* from 0.70 to 0.98 increases pCO_2_ by 46.36 μatm in this model (range: −21.6 to 24.8 μatm, wide gray bars in Figure 3a, Table S2). This is higher than the “nutrient restoring” case in Kwon et al. (2009), but lower than their “constant export” case, consistent with our model's dynamic biological productivity and interactive biogeochemistry response.

Alternative profiles with reduced export flux through 1 km and reduced biological carbon storage result in increased atmospheric pCO_2_, and vice versa (Figure 3a, Table S3). The double exponential function has the most free parameters (four) and therefore fits the power‐law extremely well, producing small differences in atmospheric pCO_2_ (less than 2 μatm). The rational function also agrees well, but could produce larger anomalies if the reference profile's *b*‐value was further from 1.00, that is, 0.70. Stretched exponential and ballast curves produce moderate changes in atmospheric pCO_2_ but are generally smaller than, or similar to, the 0.14 changes in *b* for the power‐law curves (Figure 3a). However, the simple exponential and gamma function anomalies clearly deviate from the other simulations, with greater biological carbon concentrations and drawdown of atmospheric CO_2_ for the RFIT simulations, and the inverse for AFIT and EFD simulations. Export fluxes and remineralization are significantly different in the upper ocean for these parameterizations, which can be explained by their largely invariant attenuation rates with depth (Figure 1 insets): simple exponential and gamma parameterizations cannot have both short remineralization lengthscales in the upper ocean and long remineralization length scales in the deep ocean.

There are multiple ways to compare parametric and structural uncertainty quantitatively. Parametric uncertainty is found by varying the power‐law exponent within its plausible global range (*b* = 0.84 ± 0.14), producing absolute atmospheric pCO_2_ anomalies of 21.6–24.8 μatm (Figure 3a, Table S3). For structural uncertainty, the median change in absolute atmospheric pCO_2_ is 12.47 ± 10.67 μatm (*b*‐anomaly equivalent of 0.07 ± 0.06) across all simulations with alternate functional forms. We choose the median ± median absolute deviation so that our result is robust to large anomalies associated with simple exponential and gamma functional forms. For RFIT, AFIT, and EFD simulations separately, the medians are 15.15 ± 10.40, 10.65 ± 7.30, and 20.57 ± 15.37 μatm, respectively, giving a 15.15 ± 4.51 μatm grand median (*b*‐anomaly equivalent of 0.09 ± 0.03). Excluding profiles with largely invariant attenuation rates with depth, that is, exponential and gamma function profiles, the overall medians for RFIT, AFIT, and EFD are 10.07 ± 2.32, 7.96 ± 2.69, and 10.57 ± 1.98 μatm, respectively, with a 10.07 ± 0.50 μatm grand median (*b*‐anomaly equivalent of 0.06 ± 0.00). In summary, our results are largely robust, indicating a structural uncertainty of 10–15 μatm, roughly half of parametric uncertainty for the biological pump (22–25 μatm, *b* = 0.84 ± 0.14), analogous to a ∼0.08 change in *b*.

### 3.4 Role of Nonlinearity in the Biological Pump

Much emphasis is placed on the biological pump's effect on climate by significantly lowering atmospheric CO_2_ levels, but our exponential and gamma function simulations indicate that having a biological pump (i.e., uptake, export, and depth‐dependent remineralization) and an associated biological carbon store is not necessarily sufficient to produce atmospheric carbon drawdown of the expected magnitude, such as a ∼ 200 μatm difference between biotic and abiotic oceans (Volk & Hoffert, 1985). To understand what aspects of the biological pump are important for significantly lowering atmospheric CO_2_, we ran a simulation (“NOPOM”) that represents a hypothetical ocean with no particulate organic matter export. Instead, biological production is channeled into dissolved organic matter that is remineralized near the surface.

Atmospheric pCO_2_ in NOPOM increases 165.4 μatm (Table S2) with respect to our reference power‐law: slightly less outgassing than Volk and Hoffert (1985), but the NOPOM ocean does have biological activity and a small biogenic carbon store. This is roughly twice as large as the outgassing resulting from the use of a simple exponential remineralization profile fit to the reference power‐law curve in AFIT and EFD simulations (70.3 and 92.6 μatm), despite these simulations supporting significant 1 km export fluxes (1.460 and 1.238 PgC y^−1^, only 20% less than the reference power‐law) as well as large stores of biological carbon (1,830 and 1,900 PgC, compared to 176 PgC for NOPOM). Thus, only about half of the biological pump's effect on atmosphere‐ocean carbon drawdown (∼80 μatm) can be attributed to export of particulate organic matter and biological carbon storage (Figure 3b).

The remaining ∼80 μatm drawdown in atmospheric carbon content is due to the change in shape of remineralization curves between a biological pump represented by AFIT and EFD exponential curves compared to a biological pump represented by the reference power‐law profile. Exponential profiles have a constant rate of change of remineralization, or attenuation of the sinking particle flux, with depth (Figures 1c and 1d, insets), which results in the majority of the sinking particle flux from the surface ocean being remineralized in the upper 2 km. Export fluxes through this horizon are 0.204 and 0.140 PgC y^−1^. Alternatively, attenuation for the power‐law curve decreases significantly with depth, leading to a substantial 2 km export flux of 0.802 PgC y^−1^. Thus, for AFIT and EFD exponential profiles, there is much less abyssal biological carbon storage to act as a long‐term reservoir of atmospheric CO_2_, whereas rapidly decreasing attenuation in the reference power‐law supports long‐term biological carbon storage.

In other words, decreasing upper ocean particle attenuation, or increasing remineralization lengthscale with depth, appears to be equally important for air‐sea carbon partitioning as export and storage of biological carbon (Figure 3b).

## 4 Discussion and Conclusions

Atmospheric CO_2_ levels are intimately tied to the strength of the ocean's biological pump (Ito & Follows, 2005; Volk & Hoffert, 1985). The challenge of measuring particulate fluxes via sediment traps, optical proxies, or geochemical methods (Berelson, 2001; Guidi et al., 2015; Henson et al., 2012; Honjo et al., 2008; Martin et al., 1987; Pavia et al., 2019), the spatiotemporal variability of fluxes, and the complexity of the governing mechanisms introduce uncertainty into representation of the biological pump in ocean biogeochemistry, ecosystem, and climate models. We explored the impact of structural uncertainty—remineralization profile shape—on atmosphere‐ocean carbon partitioning, using seven mechanistically distinct functional forms of particulate organic matter flux that capture observational spread equivalently well (Cael & Bisson, 2018). In our model, a 0.14 change in the power‐law exponent, *b*, results in a 22–25 μatm change in atmospheric pCO_2_, indicating that the structural uncertainty revealed by our simulations of 10–15 μatm is equivalent to ∼0.08 change in the global *b* value. Thus structural uncertainty is roughly half the size of parametric uncertainty, making it a substantial one‐third contribution to our overall estimate of total uncertainty (the sum of structural and parametric uncertainties) in understanding the biological pump. In addition, our result is in the upper range of the 5–15 μatm uncertainty associated with regional variation in *b* (Wilson et al., 2019).

Historically, the focus has been on remineralization length*scale* (Kwon et al., 2009), but our results, indicating that vertical gradient in attenuation is a first‐order control on climate, imply that multiple length*scales* of attenuation are critical to the biological pump's global impact. Thus, not only is the existence of a biological pump that maintains interior ocean biological carbon stores a key factor in the biological pump's modulation of atmospheric CO_2_ levels (Volk & Hoffert, 1985), but also a significant decrease of attenuation with depth is necessary to achieve the full amount of drawdown usually attributed to the biological pump (Figure 3b). Even when the exponential profiles' parameters are determined by matching the e‐folding remineralization depth of the reference power‐law curve (Kwon et al., 2009), the result is still large atmospheric pCO_2_ anomalies caused by largely invariant attenuation rates with depth.

Our study evaluates structural uncertainty in the ocean's biological pump in a systematic way. Although previous studies have compared individual, or a subset, of the alternative remineralization curves used here (e.g., Yamanaka & Tajika, 1996; Gehlen et al., 2006; Kriest & Oschlies, 2008; Schwinger et al., 2016; Gloege et al., 2017; Niemeyer et al., 2019; Kriest et al., 2020) with a focus on minimizing model‐observational differences, none has attempted to evaluate this structural uncertainty by just changing the shape of the remineralization profile, which we do here by comparing six alternative functional forms statistically fit in three different ways to a reference power‐law profile. Despite these profile choices resulting in non‐negligible differences in ocean biogeochemical distributions (Aumont et al., 2017; Kriest et al., 2012) and atmospheric CO_2_ levels (Kwon et al., 2009), comparison of model output to climatological data (Boyer et al., 2018; Garcia et al., 2018) does not significantly change (Figure S8), such that all the curves still quantitatively reproduce the observations to a similar degree.

As Earth System Models continue to rely on simple biological pump parameterizations, our estimate of structural uncertainty underscores the importance of research aimed at improving the basic mechanistic characterization of the biological pump (Boyd et al., 2019), and particularly the depth‐dependence or evolution of these mechanisms. One such improvement is to consider the spectrum of sinking particle properties, such as size (Schwinger et al., 2016; Niemeyer et al., 2019), sinking speeds (Kriest & Oschlies, 2008) or material lability (Aumont et al., 2017), and how they affect export fluxes. These studies often derive components that rely on upper and lower incomplete gamma functions, as well as gamma distributions, but ultimately do not produce gamma function flux profiles. The Rothman and Forney (2007) profile (Equation S6) is a special case of the upper incomplete gamma function (where the order, *a* = 0). However, statistical fits of integer orders of the upper incomplete gamma function where *a* > 0 to the reference power‐law (*b* = 0.84) are poor (See Figure S1, including the simple exponential curve, which is proportional to an upper incomplete gamma function of order *a* = 1), and as stand‐alone remineralization parameterizations may include particle classes whose remineralization profiles may not exist in the ocean. On the other hand, a more general three‐parameter upper incomplete gamma function parameterization, *C*_*g*_Γ(*a*_*g*_, *z*/*ℓ*_*g*_), fits the Martin Curve very well with *a*_*g*_ ≈ −0.8 (Figure S1), and would correspond to a constant‐sinking reactivity continuum model (Aumont et al., 2017) with a power‐law reactivity distribution. However, reactivity continuum models do not describe reactivity using a power law and instead use lighter‐tailed distributions such as the gamma (Boudreau & Ruddick, 1991), beta (Vähätalo et al., 2010), or log‐normal distribution (Forney & Rothman, 2012). Thus we did not include these additional profiles in our biological pump structural error ensemble as there is not a justifiable basis for *a* > 1, nor a plausible mechanism for *a* < 0, unlike the six alternative remineralization curves presented.

A better process‐based understanding is critical to choosing between these parameterizations based on their mechanistic underpinnings and thus reducing structural uncertainty, because empirical fits to flux measurements alone cannot currently do so (Cael & Bisson, 2018; Gehlen et al., 2006). Indeed, there are also no guarantees that more extensively sampled ocean nutrient distributions are able to distinguish between the performance of idealized and more explicit remineralization schemes either (Niemeyer et al., 2019; Schwinger et al., 2016).

In our simulations, the parameterizations were forced to be as similar as possible with regard to the three different criteria (minimizing misfit error or matching the reference e‐folding depth of remineralization), but functional forms based on different processes will have different sensitivities to temperature and other phenomena, and therefore will produce divergent projections and different climate feedbacks. Furthermore, each alternative functional form will be associated with its own parametric uncertainty. Unfortunately, significantly less is known about the natural range of parameters associated with the alternative remineralization profiles in the real ocean, because they have not been used as widely as the Martin Curve.

There are other factors that could affect the distribution, export, and depth‐dependent remineralization of sinking particles, and therefore ocean carbon sink/atmospheric CO_2_ sensitivity, that we held the same between simulations. For example, our assumption of a closed carbon cycle with no sediment burial or riverine fluxes may underestimate the biological pump effect on atmospheric CO_2_ for the different remineralization profiles by four to seven times (Roth et al., 2014) on timescales of 10–100 thousand years. Between different models, the overall strength of the deep ocean carbon store may be more dependent on remineralization profile parameters than on different ocean circulations, although circulation impact on upper ocean production would modify the overall relationships shown here (Kriest et al., 2020; Romanou et al., 2014). Vertical grid resolution and numerical diffusion might also result in changes to the ocean carbon sink (Kriest & Oschlies, 2011), although again these changes may not manifest in the short timespan that many more complex coupled ocean‐ecosystems are integrated for (Kwon et al., 2009; Schwinger et al., 2016). Despite these challenges, it would be valuable to compare these different functional forms within state‐of‐the‐art Earth System Models, either directly or via implied remineralization profile shape, to improve confidence in projections involving biosphere‐climate interactions.

## Conflict Of Interests

The authors declare that there are no conflict of interests.

## Supporting information

Supporting Information S1

## Data Availability

Model input, code, and output processing routines can be accessed via http://bit.ly/lauderdale-cael-export-profile-shape (Lauderdale & Cael, 2021).
