# Supplementary material for: Impact of Remineralization Profile Shape on the Air‐Sea Carbon Balance
Source: Geophys Res Lett. 2021 Apr 9;48(7):e2020GL091746. doi: 10.1029/2020GL091746 (PMC8243937; doi:10.1029/2020GL091746)
Supplement: Supplementary file 1 — Supporting Information S1 [file GRL-48-e2020GL091746-s001.pdf]

Supporting Information for "Impact of remineralization profile shape on the air-sea carbon balance"

Jonathan Maitland Lauderdale[1] and B. B. Cael[2]

[1]Department of Earth, Atmospheric and Planetary Sciences, Massachusetts Institute of Technology, Cambridge, MA, USA.

[2]Ocean Biogeosciences, National Oceanography Centre, Southampton, UK.

Corresponding authors: JML (jml1@mit.edu) & BBC (cael@noc.ac.uk)

Contents of this file

1. Text "Introduction", "Alternative remineralization profile shapes", and "Supporting simulation results".
2. Figures S1 to S8.
3. Tables S1 to S3.

Introduction

This Supplementary Information contains supporting materials and methods, figures for individual model simulations presented in aggregated form in the main text, as well as tables containing additional globally-aggregated values and anomalies. Model input,

code, and output processing routines can be accessed via `http://bit.ly/lauderdale-cael-export-profile-shape` (Lauderdale & Cael, 2021)

Alternative remineralization profile shapes.

Here we outline the derivation and assumptions behind six different remineralization profiles. Assuming timescales of sinking ($\leq$1 month) are shorter than transport by ocean circulation ($\sim$1 year), biological material can be approximated as instantaneously redistributed and remineralized in the vertical. $f(z)$ is the fraction of the flux of particulate organic matter from a productive layer near the surface (Buesseler et al., 2020) sinking through the depth horizon z [m].

The most basic curve is a "simple exponential", assuming constant first-order remineralization kinetics and velocity (Banse, 1990; Dutkiewicz et al., 2005; Marsay et al., 2015; Gloege et al., 2017):

$$f_e(z) = C_e e^{-\frac{z}{\ell_e}}, \tag{1}$$

where and C_e is a scaling coefficient and ℓ_e [m] is a characteristic lengthscale-the ratio of remineralization timescale and sinking speed.

Including an additional flux of refractory material c to increase the net sinking flux produces the "ballast" model (Armstrong et al., 2001; Gloege et al., 2017):

$$f_b(z) = C_b e^{-\frac{z}{\ell_b}} + c, \tag{2}$$

while explicitly considering the transformation of labile particles with a characteristic lengthscale, ℓ_{d1}, and more refractory material with a characteristic length scale, ℓ_{d2} results

in a "double exponential" profile (Lutz et al., 2002):

$$f_d(z) = C_{d1}e^{-\frac{z}{\ell_{d1}}} + C_{d2}e^{-\frac{z}{\ell_{d2}}}. \tag{3}$$

Relaxing the assumption of constant remineralization timescale, and considering a decrease in the rate of remineralization as labile material is preferentially transformed and refractory material is left behind (as in Eq. 1), leads to a "stretched exponential":

$$f_s(z) = C_s e^{-z^{(1-s)}}, \tag{4}$$

where s is a scale factor between 0–1, for example, if marine particles are degrade similarly to marine sediments, $s \approx 0.95$ (Middelburg, 1989; Cael & Bisson, 2018). A three-parameter stretched exponential with z normalized by z_o is used in many applications. However, fitting z_o and s simultaneously is ill-conditioned, i.e. parameter values are not identifiable, so we have used the simpler two-parameter stretched exponential function, which still provides fits well within global particle flux uncertainty (see Fig. 1).

Second-order degradation kinetics leads to a rational form (Suess, 1980):

$$f_r(z) = \frac{C_r}{z + a} \tag{5}$$

where C_r [m] is determined by remineralization and sinking while a [m] is determined by remineralization, sinking, and the initial flux (Cael & Bisson, 2018).

One can model sinking particles as heterogeneous media containing organic carbon, ballast minerals, and heterotrophic bacteria where remineralization slows with time (Rothman & Forney, 2007). This translates to an upper incomplete "gamma" function

curve, $\Gamma(a, x)$, of zeroth order (Cael & Bisson, 2018):

$$f_g(z) = C_g \Gamma\left(0, \frac{z}{\ell_g}\right), \tag{6}$$

where ℓ_g [m] relates to sinking speed and intraparticle bacterial concentration (Rothman & Forney, 2007; Cael & Bisson, 2018). The upper incomplete gamma function can take any value for order a, and is a component of other remineralization profile studies (e.g. Aumont et al 2017, Kriest and Oschlies 2008). For positive integer values of a, when $a = 1$ then $C_g\Gamma(1, z/\ell_g) = C_g e^{-z/\ell_g}$ (which is the simple exponential profile), and if $a = 2$ then there is a recursion relation where $C_g\Gamma(2, z/\ell_g) = C_g\Gamma(1, z/\ell_g) + C_g z/\ell_g e^{-z/\ell_g}$, and so on. We repeated our statistical fit to the reference power law curve for upper incomplete gamma functions with orders of a between 0–3 (Figure S1). The best fits to the reference power-law (b=0.84) are in fact when $a = 0$ regardless of minimizing absolute or relative errors (AFIT or RFIT), and also in the EFD case. The simple exponential case, equivalent to $a = 1$, is a poor fit to the power-law for all three cases, and the dissimilarity increases where $a > 1$. Indeed, if $a = 2$ or 3 then attenuation increases with depth in the upper ocean, which is uncharacteristic of sinking particle observations and the Martin Curve, and the existence of a particle class whos flux profile would depend on $C_g z/\ell_g e^{-z/\ell_g}$ is unlikely. Thus, we do not run simulations where $a > 1$, which do not correspond to a plausible mechanism for sinking particle remineralization.

A more general three-parameter upper incomplete gamma function parameterization $C_g\Gamma(a_g, z/\ell_g)$ fits the Martin curve very well with $a_g \approx -0.8$ (Figure S1), and would correspond to a constant-sinking reactivity continuum model (Aumont et al., 2017) with a power-law reactivity distribution. However, reactivity continuum models do not a describe

reactivity using a power law, and instead use lighter-tailed distributions such as the gamma (Boudreau & Ruddick, 1991), beta (Vähätalo et al., 2010), or log-normal distribution (Forney & Rothman, 2012) that do not permit closed-form solutions. Again, we do not consider this parameterization as a plausible alternative to the Martin Curve for this reason.

Finally, we note that the "power-law" (Eq. 1) assumes either an increase in sinking speed with depth or a decrease in remineralization rate (Kriest & Oschlies, 2011; Cael & Bisson, 2018).

Coefficients derived by non-linear statistical fit of profiles given by Eqn. S1–S6 to the reference power-law curve are given in Table S1.

Supporting simulation results:

Fig. S2 shows steady-state zonal averages for the power-law simulations where b=0.84±0.14. Particulate organic carbon fluxes in the ocean interior increase when the power-law exponent decreases to 0.70 (Fig. S2, left column) and decrease when the power-law exponent is increased to 0.98 (Fig. S2, right column). The negative feedback between nutrient availability and biological production of particles (e.g. global experiments in Kriest & Oschlies, 2011) can be seen in the surface export flux anomalies (Fig. S2d, f). More efficient export and lower rates of upper ocean remineralization leads to a decrease in recycled nutrient availability, and therefore less overall biological productivity, and lower shallow particulate organic carbon flux when b is reduced (also see globally-integrated community production, ΔB_C, and integrated export fluxes through the deepest annual mixed layer, ΔE_{mld}, values in Fig. S3a and Table S2). However, reduced shallow export

across the deepest mixed layer depth is compensated by lower upper ocean flux attenuation, due to reduced exponent value, resulting instead in enhanced export flux anomalies integrated at 1 km depth. Nutrient availability increases when the remineralization profile is more attenuating in the upper ocean, driving enhanced shallow particulate production and export, but this flux is quickly depleted by the same remineralization profile attenuation, resulting in lower interior ocean export fluxes to the deep ocean.

Biological carbon concentration (C_{bio}) integrates these export fluxes, so that when interior ocean export increases, the deep ocean biological carbon store increases (Fig. S3b), particularly in the Southern Ocean (Fig. S2g), and the deep North Pacific (Fig. S2j), but decreases in the North Atlantic (Fig. S2g) and vice versa (Fig. S2f, i, l) (Kwon & Primeau, 2006; Kriest & Oschlies, 2011; Kriest et al., 2012; Romanou et al., 2014).

The deep ocean store of biological carbon is directly linked to air-sea carbon partitioning, thus greater ΔC_{bio} indicates uptake of atmospheric carbon by the ocean, and pCO_2 declines. Conversely, when ΔC_{bio} decreases, that carbon outgasses from the ocean causing atmospheric pCO_2 to increase (Table S2).

Fig. S4 shows zonally-averaged anomalies with respect to the reference power-law of export fluxes for the three sets of parameter values and the six different functional forms of remineralization profile. Anomalies largely have an inverse surface-deep ocean contrast, which is captured by the differences in fluxes through the deepest annual mixed layer depth (ΔE_{mld}, Table S3) for the surface ocean changes in particulate export, and fluxes though the 1 km depth horizon (ΔE_{1km}, Table S3) for the deep ocean changes in particulate export. However, the ballast functional form (Eq. S2) has a more complex distribution of

particulate flux anomalies in surface, intermediate, and deep waters associated with the additional refractory flux c, which becomes important when the exponentially decaying labile portion of the sinking flux becomes attenuated to low levels.

Fig. S5–S7 shows Atlantic and Pacific Ocean zonally-averaged anomalies with respect to the reference power-law of the concentration of biological carbon (ΔC_{bio}, Table S3), which integrates the vertical flux and remineralization of particulate organic matter. Again, anomalies largely have an inverse surface-deep ocean contrast, with similar sign anomalies in the older upwelling waters of the Southern Ocean and deep North Pacific, in contrast to the youngest waters in the North Atlantic (Kwon & Primeau, 2006; Kriest & Oschlies, 2011; Kriest et al., 2012; Romanou et al., 2014). The deep ocean store of C_{bio} is inversely proportional to atmospheric CO_2 content (Table S3).

Finally, Fig. S8 shows a comparison of model phosphate fields with the World Ocean Atlas climatology (WOA2018, Boyer et al., 2018; Garcia et al., 2018) for the 21 simulations with different remineralization profiles. Despite reorganization of carbon and nutrient concentrations, comparison of model output to climatological data does not significantly change, underscoring that although these profile choices result in non-negligible differences in ocean biogeochemical distributions (Kriest et al., 2012; Aumont et al., 2017) and atmospheric CO_2 levels (Kwon et al., 2009), the differences are small enough that all the curves still quantitatively reproduce the observations to a similar degree.

References

Armstrong, R. A., Lee, C., Hedges, J. I., Honjo, S., & Wakeham, S. G. (2001). A new, mechanistic model for organic carbon fluxes in the ocean based on the quantitative association of POC with ballast minerals. *Deep Sea Res. Part II*, *49*(1), 219–236. doi: 10.1016/S0967-0645(01)00101-1

Aumont, O., van Hulten, M., Roy-Barman, M., Dutay, J. C., Éthé, C., & Gehlen, M. (2017). Variable reactivity of particulate organic matter in a global ocean biogeochemical model. *Biogeosciences*, *14*(9), 2321–2341. doi: 10.5194/bg-14-2321-2017

Banse, K. (1990). New views on the degradation and disposition of organic particles as collected by sediment traps in the open sea. *Deep Sea Research Part A. Oceanographic Research Papers*, *37*(7), 1177–1195. doi: 10.1016/0198-0149(90)90058-4

Boudreau, B. P., & Ruddick, B. R. (1991). On a reactive continuum representation of organic matter diagenesis. *American Journal of Science*, *291*(5), 507. doi: 10.2475/ajs.291.5.507

Boyer, T. P., Garcia, H. E., Locarnini, R. A., Zweng, M. M., Mishonov, A. V., Reagan, J. R., ... Smolyar, I. V. (2018). *World Ocean Atlas 2018.* Dataset. Retrieved 3/3/2021, from https://accession.nodc.noaa.gov/NCEI-WOA18

Buesseler, K. O., Boyd, P. W., Black, E. E., & Siegel, D. A. (2020). Metrics that matter for assessing the ocean biological carbon pump. *Proc. Nat. Acad. Sci.*, *117*(18), 9679. doi: 10.1073/pnas.1918114117

Cael, B. B., & Bisson, K. (2018). Particle flux parameterizations: Quantitative and mechanistic similarities and differences. *Front. Mar. Sci.*, *5*, 395. doi: 10.3389/

fmars.2018.00395

Dutkiewicz, S., Follows, M. J., & Parekh, P. (2005). Interactions of the iron and phosphorus cycles: A three dimensional model study. *Global Biogeochem. Cycles*, *19*. doi: 10.1029/2004GB002342

Forney, D. C., & Rothman, D. H. (2012). Common structure in the heterogeneity of plant-matter decay. *Journal of The Royal Society Interface*, *9*(74), 2255–2267. doi: 10.1098/rsif.2012.0122

Garcia, H. E., Weathers, K., Paver, C. R., Smolyar, I., Boyer, T. P., Locarnini, R. A., ... Reagan, J. R. (2018). World Ocean Atlas 2018, volume 4: Dissolved inorganic nutrients (phosphate, nitrate and nitrate+nitrite, silicate). In A. Mishonov (Ed.), *NOAA Atlas NESDIS 84* (p. 35pp).

Gloege, L., McKinley, G. A., Mouw, C. B., & Ciochetto, A. B. (2017). Global evaluation of particulate organic carbon flux parameterizations and implications for atmospheric pCO_2. *Global Biogeochem. Cycles*, *31*(7), 1192–1215. doi: 10.1002/2016GB005535

Kriest, I., & Oschlies, A. (2011). Numerical effects on organic-matter sedimentation and remineralization in biogeochemical ocean models. *Ocean Model.*, *39*(3), 275–283. doi: 10.1016/j.ocemod.2011.05.001

Kriest, I., Oschlies, A., & Khatiwala, S. (2012). Sensitivity analysis of simple global marine biogeochemical models. *Global Biogeochem. Cycles*, *26*(2). doi: 10.1029/2011GB004072

Kwon, E. Y., & Primeau, F. (2006). Optimization and sensitivity study of a biogeochemistry ocean model using an implicit solver and in situ phosphate data. *Global*

Biogeochem. Cycles, *20*(4). doi: 10.1029/2005GB002631

Kwon, E. Y., Primeau, F., & Sarmiento, J. L. (2009). The impact of remineralization depth on the air–sea carbon balance. *Nature Geosci.*, *2*(9), 630–635. doi: 10.1038/ngeo612

Lauderdale, J. M., & Cael, B. B. (2021). *Model code, output, and analysis routines characterizing structural uncertainty in the ocean's biological pump.* Zenodo. Retrieved 3/3/2021, from `http://bit.ly/lauderdale-cael-export-profile-shape` doi: 10.5281/zenodo.4578678

Lutz, M., Dunbar, R., & Caldeira, K. (2002). Regional variability in the vertical flux of particulate organic carbon in the ocean interior. *Global Biogeochem. Cycles*, *16*(3), 11-1–11-18. doi: 10.1029/2000GB001383

Marsay, C. M., Sanders, R. J., Henson, S. A., Pabortsava, K., Achterberg, E. P., & Lampitt, R. S. (2015). Attenuation of sinking particulate organic carbon flux through the mesopelagic ocean. *Proc. Nat. Acad. Sci.*, *112*(4), 1089. doi: 10.1073/pnas.1415311112

Middelburg, J. J. (1989). A simple rate model for organic matter decomposition in marine sediments. *Geochim. Cosmochim. Acta*, *53*(7), 1577–1581. doi: 10.1016/0016-7037(89)90239-1

Romanou, A., Romanski, J., & Gregg, W. W. (2014). Natural ocean carbon cycle sensitivity to parameterizations of the recycling in a climate model. *Biogeosciences*, *11*(4), 1137–1154. doi: 10.5194/bg-11-1137-2014

Rothman, D. H., & Forney, D. C. (2007). Physical model for the decay and preservation

of marine organic carbon. *Science*, *316*(5829), 1325. doi: 10.1126/science.1138211

Suess, E. (1980). Particulate organic carbon flux in the oceans—surface productivity and oxygen utilization. *Nature*, *288*(5788), 260–263. doi: 10.1038/288260a0

Vähätalo, A. V., Aarnos, H., & Mäntyniemi, S. (2010). Biodegradability continuum and biodegradation kinetics of natural organic matter described by the beta distribution. *Biogeochemistry*, *100*(1), 227–240. doi: 10.1007/s10533-010-9419-4

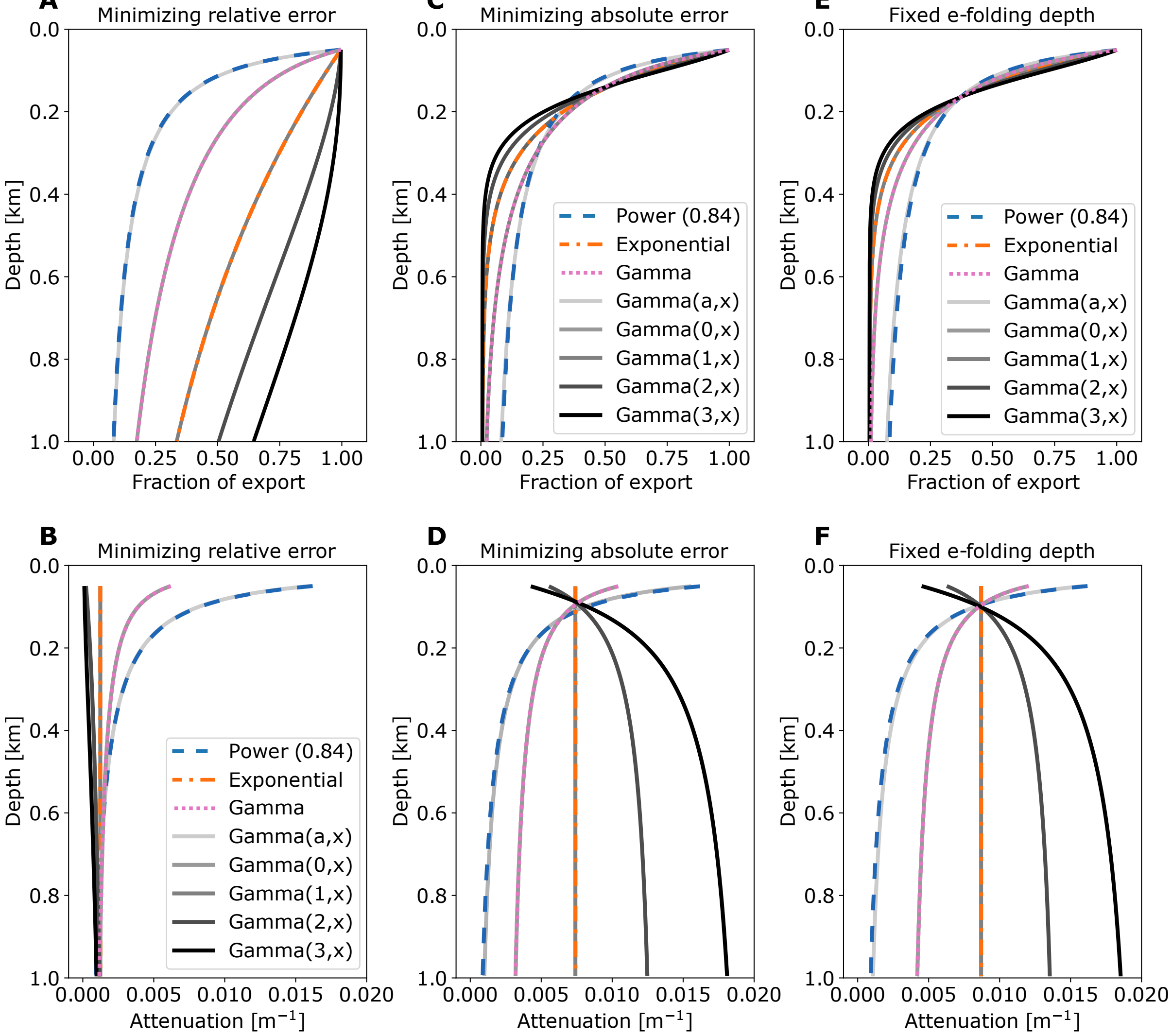

Figure S1. Fraction of sinking particulate organic matter exported from the 50 m surface layer remaining at each depth for different evaluations of the upper incomplete gamma function, $\Gamma(a, x)$, with varying values of the order a (increasing greyscale tones) and coefficients (e.g. Eqn. S6) statistically fit to the reference power-law curve (b=0.84) by (a) statistically minimizing the relative error ("RFIT"), or (c) the absolute error ("AFIT"), and (d) matching the e-folding depth scale of 164 m ("EFD"). "Gamma(a,x)" profiles are cases where the order is determined as part of a three-parameter non-linear fit giving $a \approx -0.8$. Profiles for the reference power-law (b=0.84), simple exponential, and gamma function (Eqn. S6) curves from the main text (Fig. 2) are shown in colored, dashed, lines. Plots b, d, and f show the attenuation rate of the export flux with depth $\left[\frac{1}{f}\frac{\partial f}{\partial z}, m^{-1}\right]$.

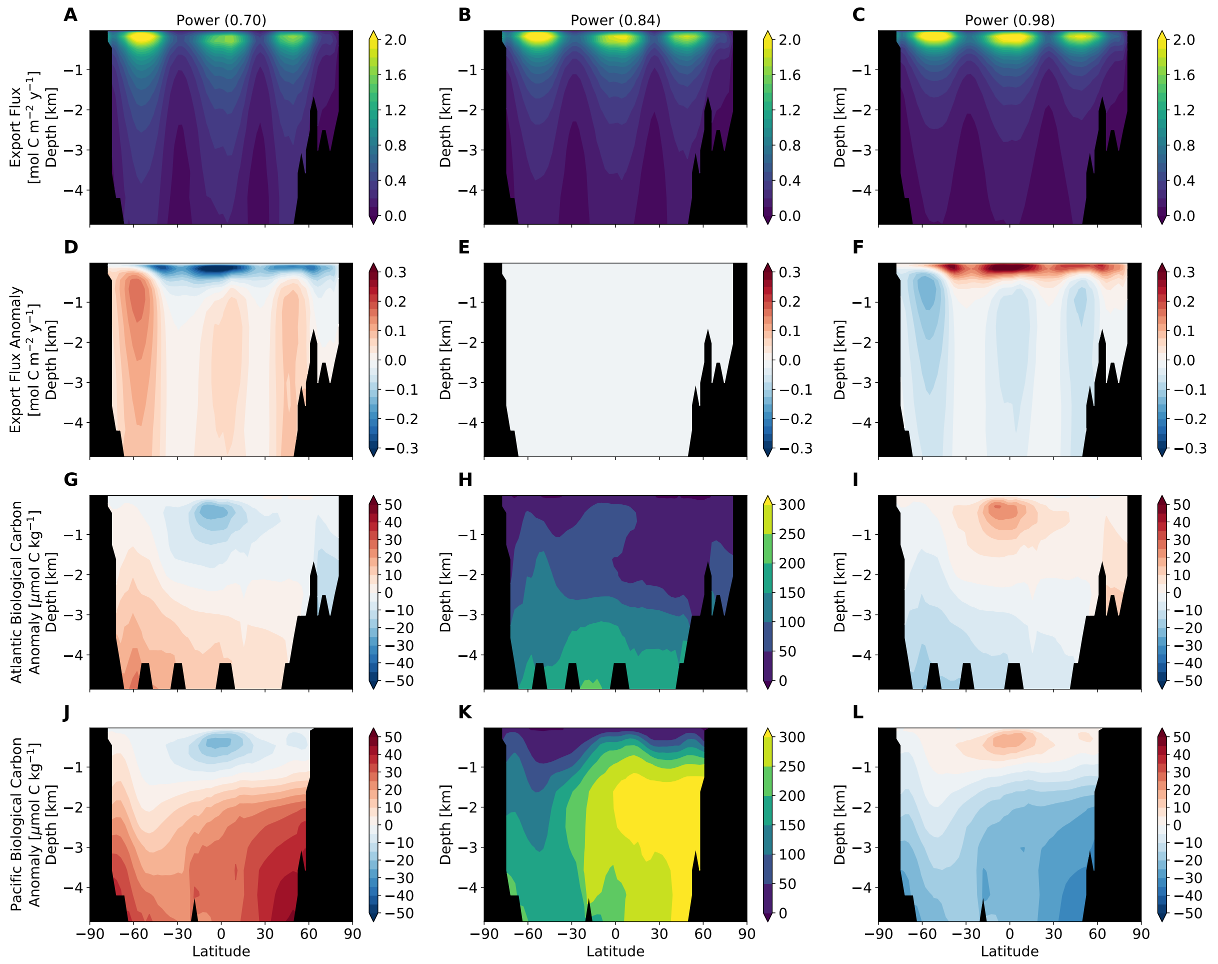

Figure S2. Zonal-average properties for power-law simulations where $b = 0.84{\pm}0.14$ (a–c) particulate organic carbon export fluxes (mol C m^{-2} y^{-1}), (d–f) anomalies of sinking particle export flux compared to the reference power-law simulations (i.e. the middle column, mol C m^{-2} y^{-1}), and biological carbon concentration (C_{bio}, μmol C kg^{-1} in the (g–i) Atlantic Ocean, and (j–l) Pacific Ocean. Reference C_{bio} concentrations are shown in the middle column, with anomalies for the decreased and increased values of the power-law exponent, b, in the left and right columns.

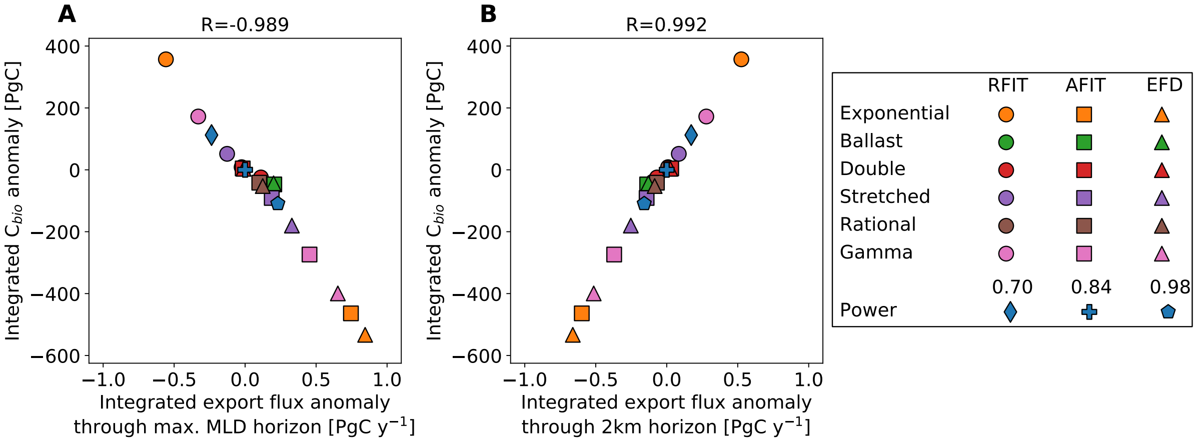

Figure S3. Change in the integrated particle export flux rate [PgC y^{-1}] passing through (a) the horizon of deepest annual mixed layer depth, and (b) the 2 km depth horizon, against integrated biological carbon reservoir anomaly [PgC], both with respect to the power-law curve where b=0.84. Three power-law simulations (b = 0.84 ± 0.14) are indicated by the blue symbols (diamond, cross, and pentagon), circle, square, and triangle symbols indicate that profile coefficients (Eq. S1–S6) were derived by minimizing the relative fit error ("RFIT"), minimizing the absolute fit error ("AFIT"), and fixing the e-folding depth of remineralization ("EFD") to the reference power-law curve. Values are shown in Table S3.

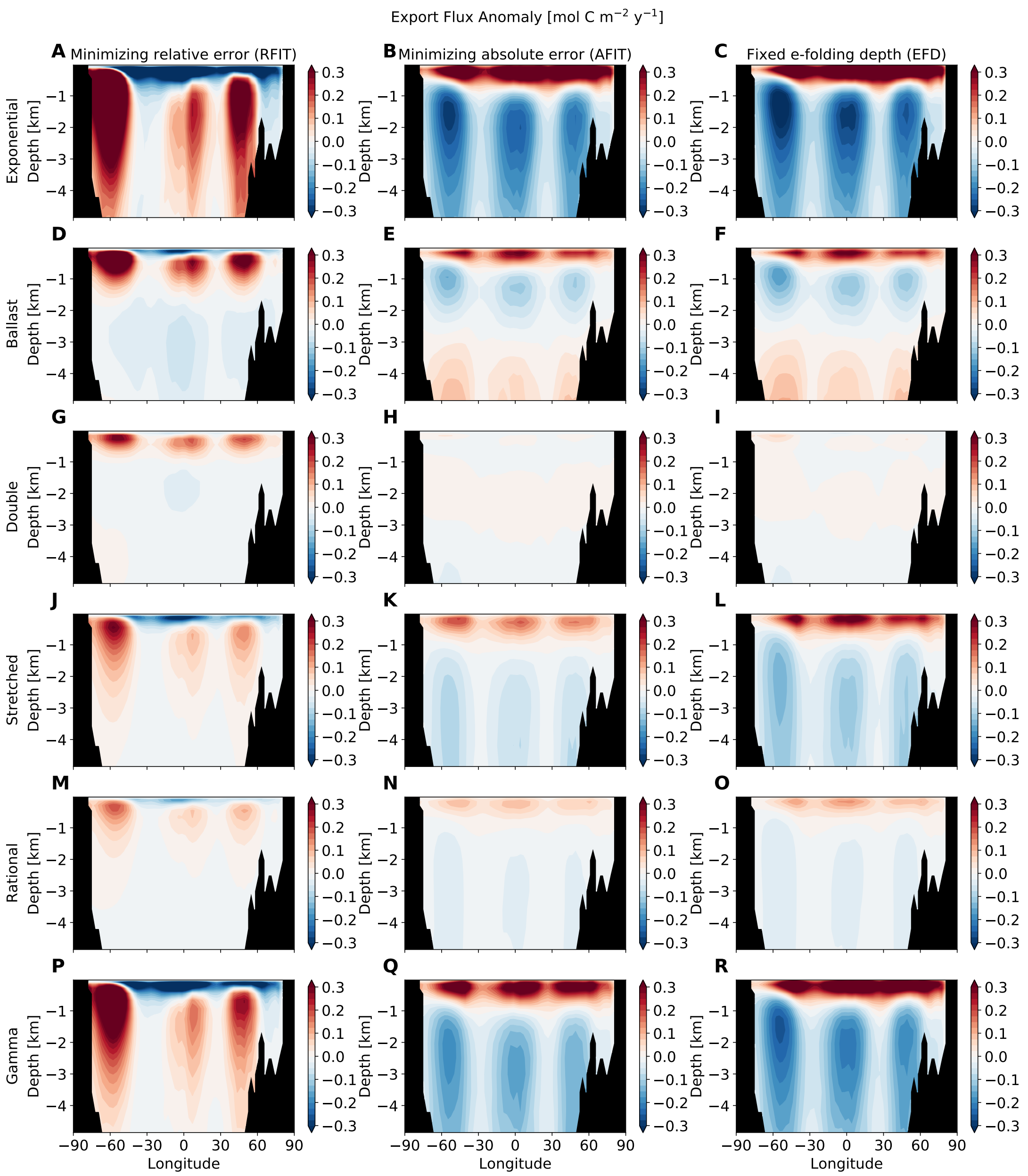

Figure S4. Zonally-averaged export flux anomaly with respect to the reference power-law curve where b=0.84, for the parameter sets where the relative error of the fit is minimized (RFIT, left column), where the absolute error of the fit is minimized (AFIT, middle column), and where the e-folding depth of remineralization is matched to the 164 m of the control curve (EFD, right column), where (a–c) is the simple exponential profile, (d–f) is the ballast profile, (g–i) is the double exponential profile, (j–l) is the stretched exponential profile, (m–o) is the rational profile, and (p–r) is the gamma profile.

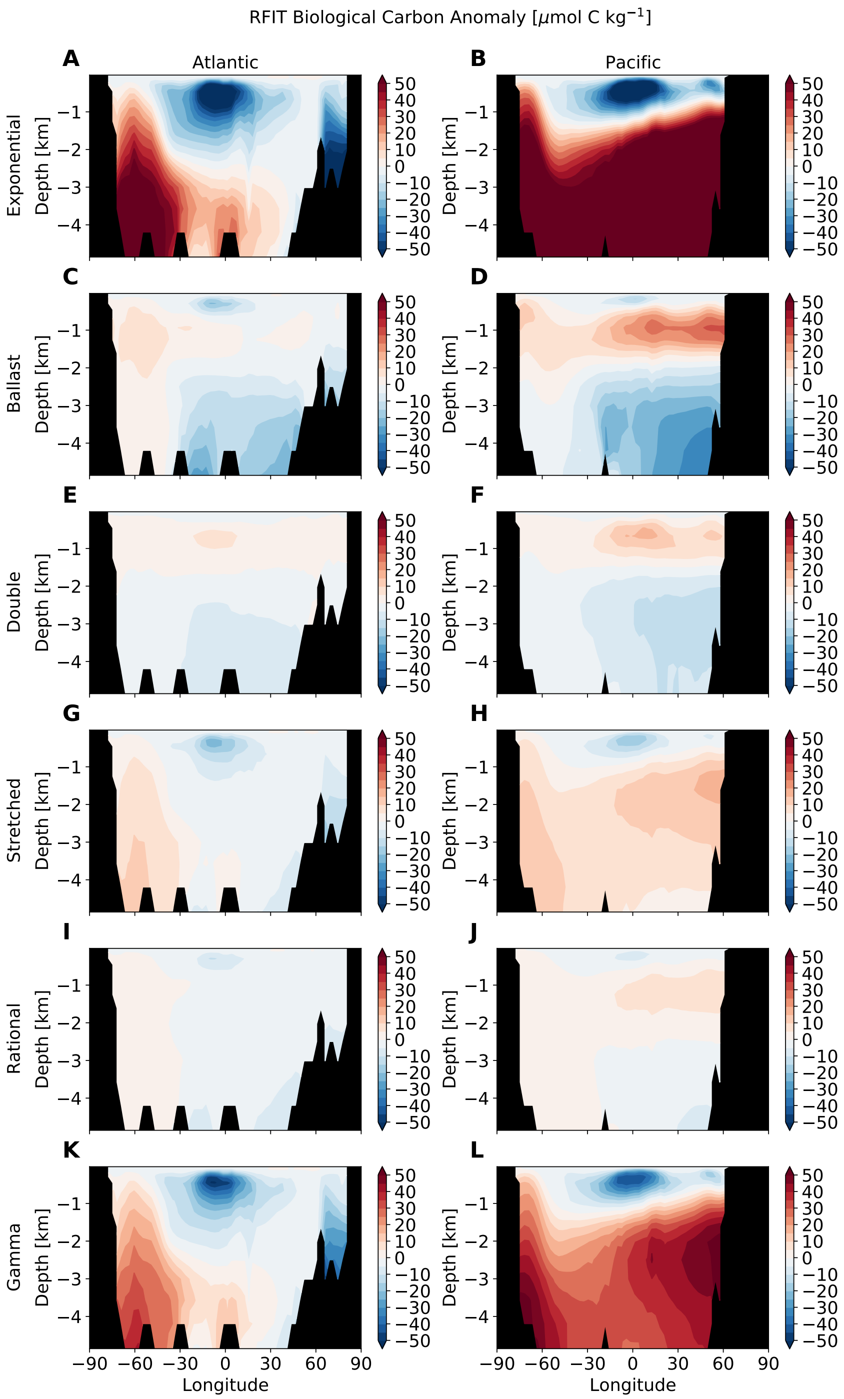

Figure S5. Zonally-averaged biological carbon (C_{bio}, μmol C kg^{-1}) anomalies with respect to the reference power-law curve for coefficients minimizing the relative error of the fit (RFIT) in the Atlantic Ocean (left column) and Pacific Ocean (right column) using (a–b) exponential, (c–d) ballast, (e–f) double exponential, (g–h) stretched exponential, (i–j) rational, and (k–l) gamma function remineralization profiles.

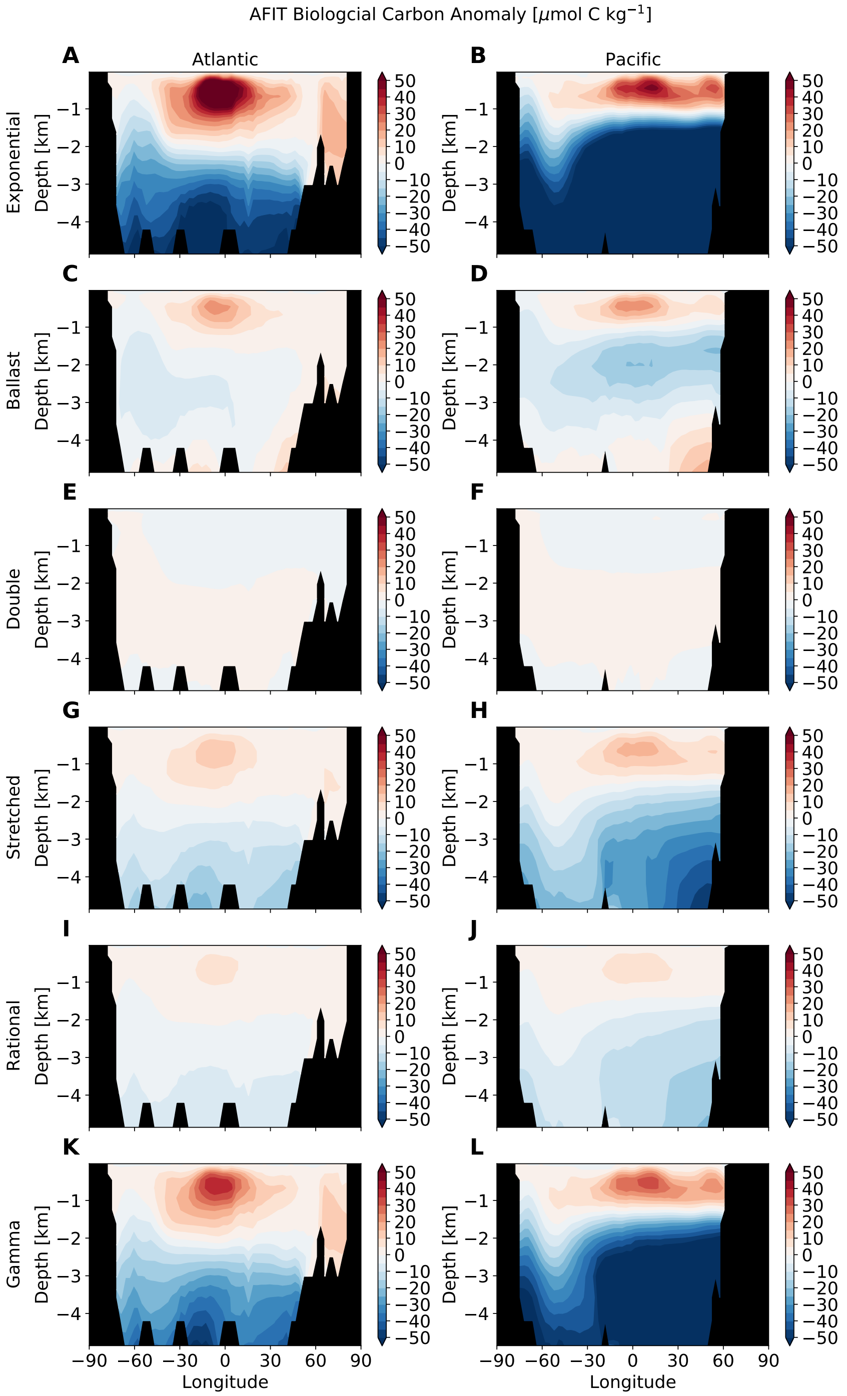

Figure S6. Zonally-averaged biological carbon (C_{bio}, μmol C kg^{-1}) anomalies with respect to the reference power-law curve for coefficients minimizing the absolute error of the fit (AFIT) in the Atlantic Ocean (left column) and Pacific Ocean (right column) using (a–b) exponential, (c–d) ballast, (e–f) double exponential, (g–h) stretched exponential, (i–j) rational, and (k–l) gamma function remineralization profiles.

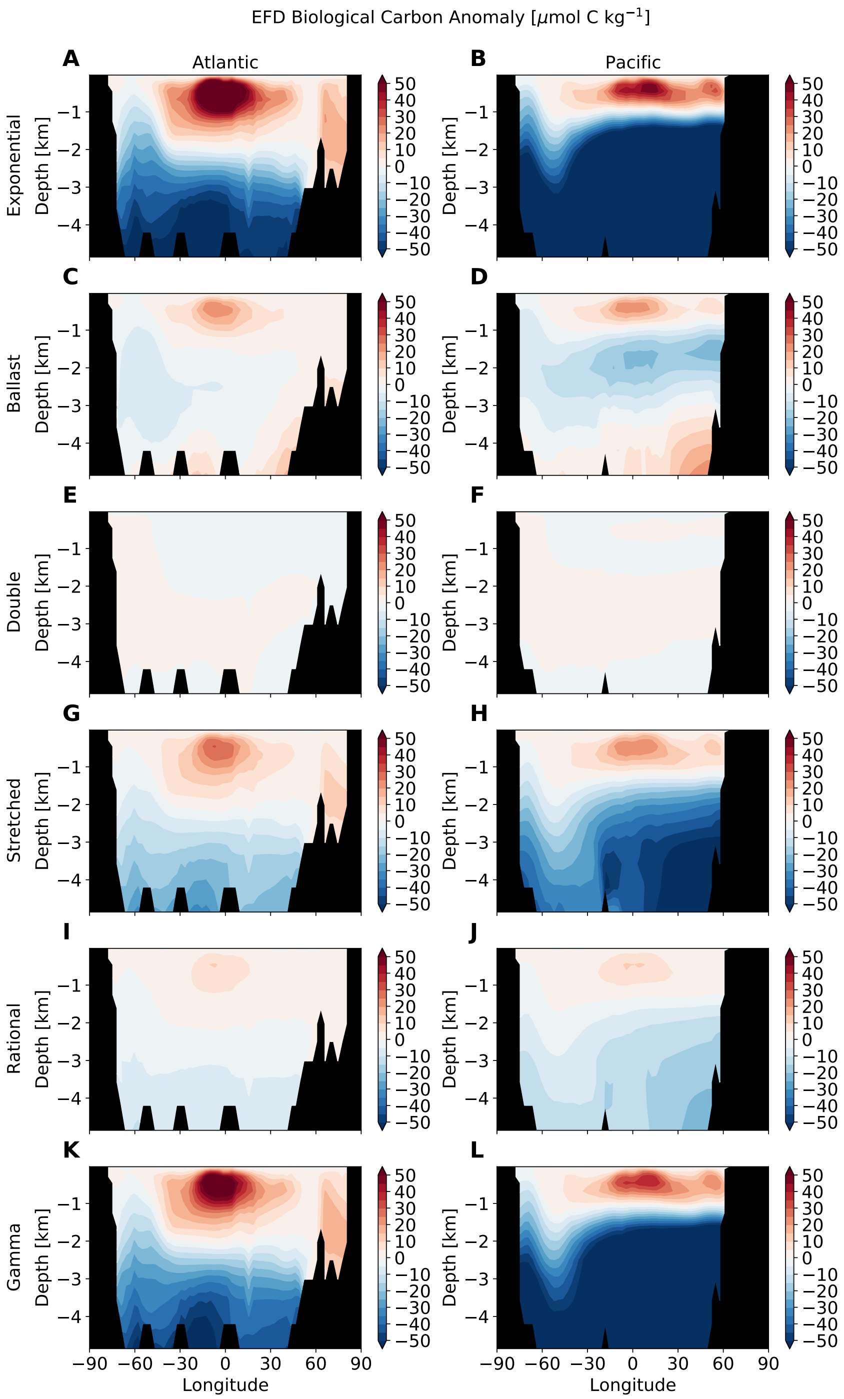

Figure S7. Zonally-averaged biological carbon (C_{bio}, μmol C kg^{-1}) anomalies with respect to the reference power-law curve for coefficients matching the 164 m e-folding depth (EFD) in the Atlantic Ocean (left column) and Pacific Ocean (right column) using (a–b) exponential, (c–d) ballast, (e–f) double exponential, (g–h) stretched exponential, (i–j) rational, and (k–l) gamma function remineralization profiles.

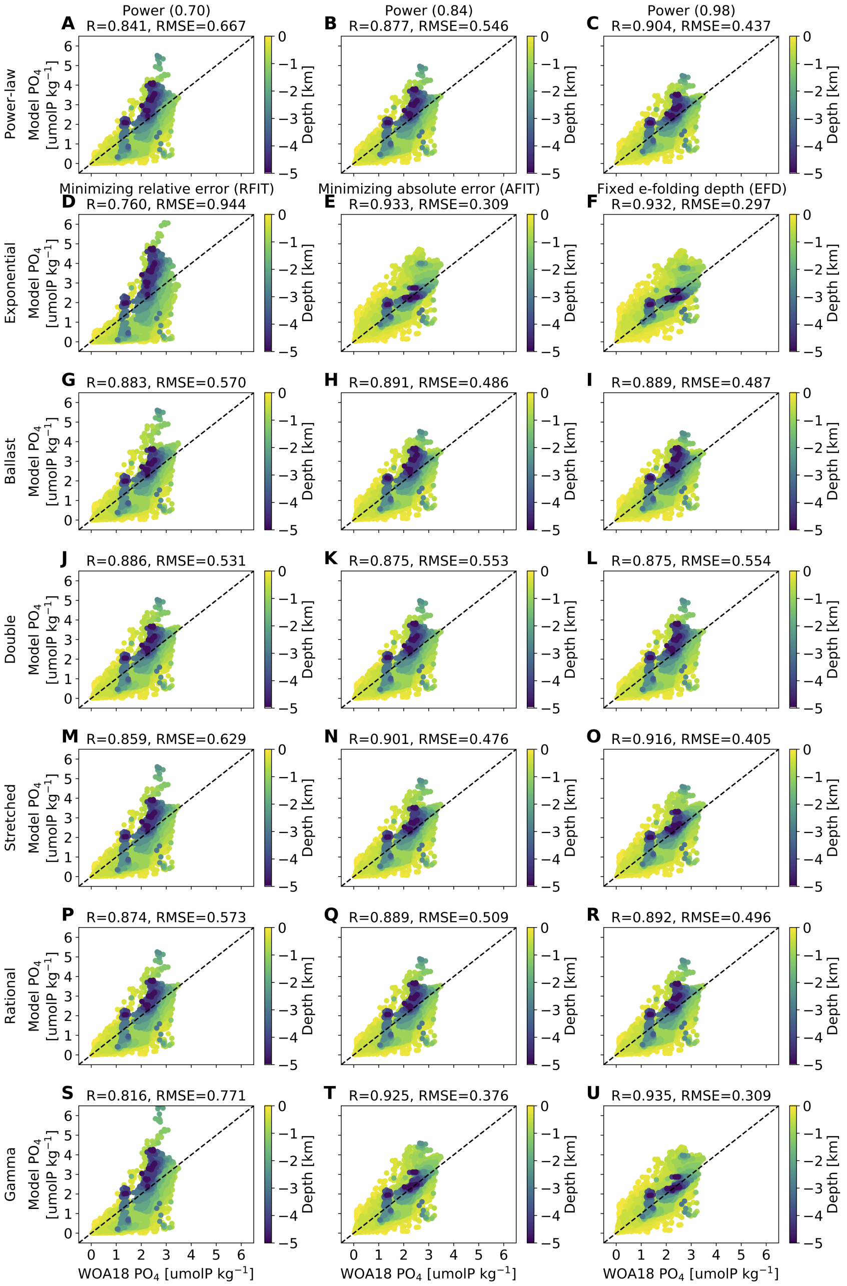

Figure S8. Model phosphate concentrations [PO_4, μmol kg^{-1}] for each remineralization profile against observations from the World Ocean Atlas 2018 (WOA18, Boyer et al., 2018; Garcia et al., 2018) colored by model depth. The black dashed line indicates a 1:1 relationship. Model-data likeness is evaluated by the Pearson correlation coefficient, R, and the root-mean-squared concentration error, $RMSE$. Although remineralization profile choices result in non-negligible differences in macronutrient distributions, the differences are small enough that all the curves still quantitatively reproduce the observations to a similar degree.

Table S1. Parameter values and fit statistics for remineralization functions (Eq. S1–S6). Each function was matched to the reference power-law (Eq. 1) with exponent b=0.84 by statistically minimizing the relative ("RFIT") or absolute ("AFIT") misfit of the curves, or by matching e-folding remineralization depth scale ("EFD"), Fig. 2b–d. Note different units of coefficients. Goodness of fit is evaluated by $\mathcal{S}$, the Standard Error of Regression (smaller numbers indicate better fit).

Shape	Parameter	Units	RFIT	AFIT	EFD
Exponential	C_e		1.059	1.451	1.548
	ℓ_e	m	871.5	134.2	114.5
	$\mathcal{S}$		1.107	0.0701	0.0700
Ballast	C_b		1.200	1.487	1.530
	ℓ_b	m	226.8	108.6	101.9
	c		0.03111	0.04159	0.04139
	$\mathcal{S}$		0.3838	0.0453	0.0440
Double Exponential	C_{d1}		1.326	1.583	1.522
	ℓ_{d1}	m	124.3	70.38	75.09
	C_{d2}		0.08668	0.1466	0.1492
	ℓ_{d2}	m	2521	1144	1170
	$\mathcal{S}$		0.1559	0.0175	0.0175
Stretched Exponential	C_s		10.88	13.91	15.81
	s		0.7776	0.7526	0.7404
	$\mathcal{S}$		0.2499	0.0260	0.0314
Rational	C_r	m	88.75	69.87	66.61
	a	m	38.75	19.87	16.61
	$\mathcal{S}$		0.1174	0.0112	0.0119
Gamma Function	C_g		0.3214	0.6003	0.7267
	ℓ_g	m	1950	419.6	300.6
	$\mathcal{S}$		0.6272	0.0499	0.0543

Table S2. Supplementary quantities for power-law remineralization simulations with exponents of b=0.70, 0.84, and 0.98, as well as the "NOPOM" simulation where there are no particulate organic matter export fluxes. Reference rates/concentrations are presented for the control power-law curve where b=0.84, while values presented for simulations where b=0.70, 0.98, and NOPOM are anomalies with respect to the control. ΔB_C is the change in globally-integrated rate of net community production, ΔE_{mld}, ΔE_{1km}, and ΔE_{2km} are the change in areally-integrated particulate organic carbon export flux through the deepest mixed layer depth, 1 km, and 2 km horizons, respectively, ΔC_{bio} is the globally-integrated change in biological carbon (evaluated as dissolved inorganic carbon minus the preformed carbon concentration), and ΔpCO_2^{atm} is the change in atmospheric CO_2 partial pressure.

		Exponent (b)			
		0.70	0.84	0.98	NOPOM
C_p	m^b	1.000	1.000	1.000	
ΔB_C	PgC y^{-1}	-5.231	29.570	5.175	39.65
ΔE_{mld}	PgC y^{-1}	-0.236	2.349	0.230	-2.349
ΔE_{1km}	PgC y^{-1}	0.141	1.749	-0.173	-1.749
ΔE_{2km}	PgC y^{-1}	0.172	0.802	-0.159	-0.802
ΔC_{bio}	PgC	112.32	2363.4	-109.30	-2187
ΔpCO_2^{atm}	μatm	-21.59	269.33	24.77	165.4

Table S3. Supplementary anomalies for alternative remineralization profile simulations. Reference power-law values (b=0.84) are given in Table S2. ΔB_C is the change in globally-integrated net community production rate, ΔE_{mld}, ΔE_{1km}, and ΔE_{2km} are the change in areally-integrated particulate organic carbon export flux through the deepest mixed layer depth, 1 km, and 2 km horizons, respectively, ΔC_{bio} is the globally-integrated change in biological carbon (evaluated as dissolved inorganic carbon minus the preformed carbon concentration), and ΔpCO_2^{atm} is the change in atmospheric CO_2 partial pressure.

Shape	Parameter	Units	RFIT	AFIT	EFD
Exponential	ΔB_C	PgC y^{-1}	-17.39	7.866	11.01
	ΔE_{mld}	PgC y^{-1}	-0.558	0.745	0.845
	ΔE_{1km}	PgC y^{-1}	0.6860	-0.2890	-0.511
	ΔE_{2km}	PgC y^{-1}	0.5250	-0.5980	-0.662
	ΔC_{bio}	PgC	357.1	-463.9	-533.3
	ΔpCO_2^{atm}	μatm	-62.94	70.28	92.59
Ballast	ΔB_C	PgC y^{-1}	-5.218	2.549	3.108
	ΔE_{mld}	PgC y^{-1}	0.124	0.205	0.201
	ΔE_{1km}	PgC y^{-1}	0.3380	-0.244	-0.292
	ΔE_{2km}	PgC y^{-1}	-0.097	-0.138	-0.130
	ΔC_{bio}	PgC	-38.40	-47.16	-43.58
	ΔpCO_2^{atm}	μatm	-12.39	10.99	12.55
Double Exponential	ΔB_C	PgC y^{-1}	-1.099	-0.2730	-0.553
	ΔE_{mld}	PgC y^{-1}	0.111	-0.017	-0.011
	ΔE_{1km}	PgC y^{-1}	0.019	-0.021	-0.010
	ΔE_{2km}	PgC y^{-1}	-0.069	0.031	0.025
	ΔC_{bio}	PgC	-24.66	4.514	3.255
	ΔpCO_2^{atm}	μatm	-1.761	-1.082	-1.821
Stretched Exponential	ΔB_C	PgC y^{-1}	-5.272	1.226	4.483
	ΔE_{mld}	PgC y^{-1}	-0.127	0.188	0.329
	ΔE_{1km}	PgC y^{-1}	0.234	0.056	-0.075
	ΔE_{2km}	PgC y^{-1}	0.085	-0.142	-0.253
	ΔC_{bio}	PgC	51.61	-90.93	-180.2
	ΔpCO_2^{atm}	μatm	-17.92	10.31	28.60
Rational	ΔB_C	PgC y^{-1}	-2.525	0.892	1.593
	ΔE_{mld}	PgC y^{-1}	-0.025	0.100	0.124
	ΔE_{1km}	PgC y^{-1}	0.120	0.000	-0.026
	ΔE_{2km}	PgC y^{-1}	0.009	-0.069	-0.085
	ΔC_{bio}	PgC	8.479	-41.63	-52.47
	ΔpCO_2^{atm}	μatm	-7.745	5.612	8.583
Gamma Function	ΔB_C	PgC y^{-1}	-11.42	4.023	8.622
	ΔE_{mld}	PgC y^{-1}	-0.330	0.454	0.653
	ΔE_{1km}	PgC y^{-1}	0.471	-0.009	-0.282
	ΔE_{2km}	PgC y^{-1}	0.279	-0.370	-0.514
	ΔC_{bio}	PgC	172.29	-273.74	-399.44
	ΔpCO_2^{atm}	μatm	-40.38	35.70	66.35
